# Supplementary figures and images for: Intellectual disability-associated gain-of-function mutations in CERT1 that encodes the ceramide transport protein CERT
Source: PLoS One. 2020 Dec 21;15(12):e0243980. doi: 10.1371/journal.pone.0243980 (PMC7751862; doi:10.1371/journal.pone.0243980)

S1 Fig

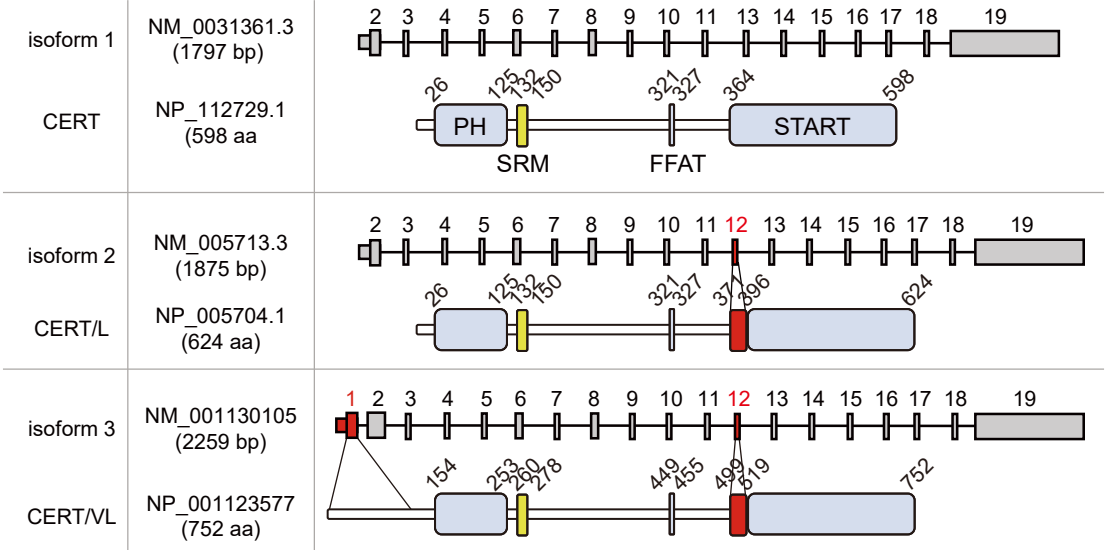

Supplement: S1 Fig — (A) The structures of CERT1 transcripts (isoforms 1–3) whose exons are indicated by gray boxes (top panel) and their protein products (CERT/L, CERT, and CERT/VL) (bottom panel). CERT has a PH domain, a SRM, a FFAT motif, and a START domain. Isoform 1 (NM_0031361.3) contains 17 exons (2–11 and 13–19) and is translated into CERT (NP_112729.1) (top panel). Isoform 2 (NM_005713.3) contains 18 exons (2–19) and is translated into CERT/L (NP_005704.1) (middle panel). Isoform 3 (NM_001130105) contains 19 exons (1–19) and is translated into CERT/VL (NP_001123577) (bottom panel). (PDF) [file pone.0243980.s001.pdf]

S2 Fig

A

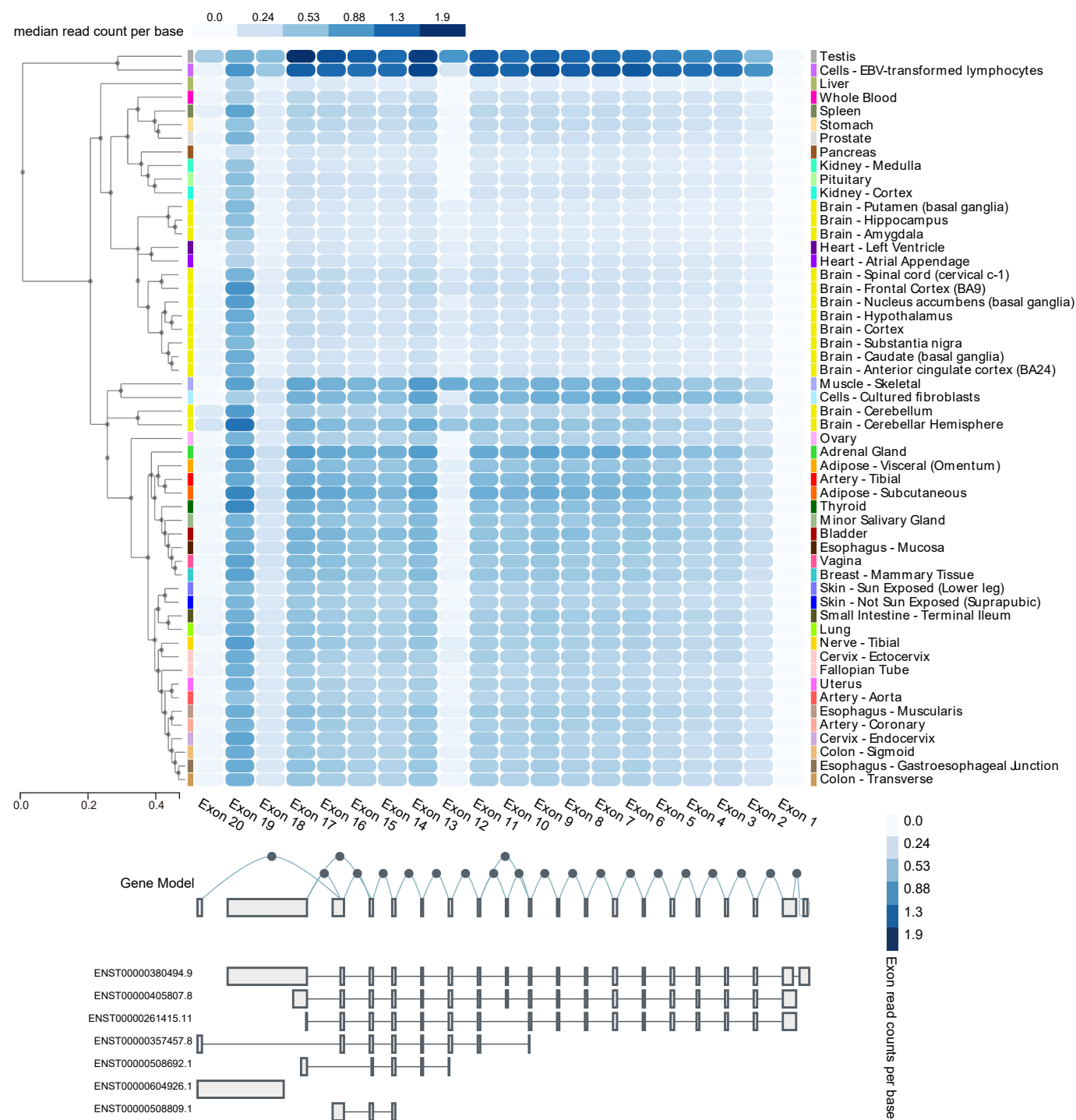

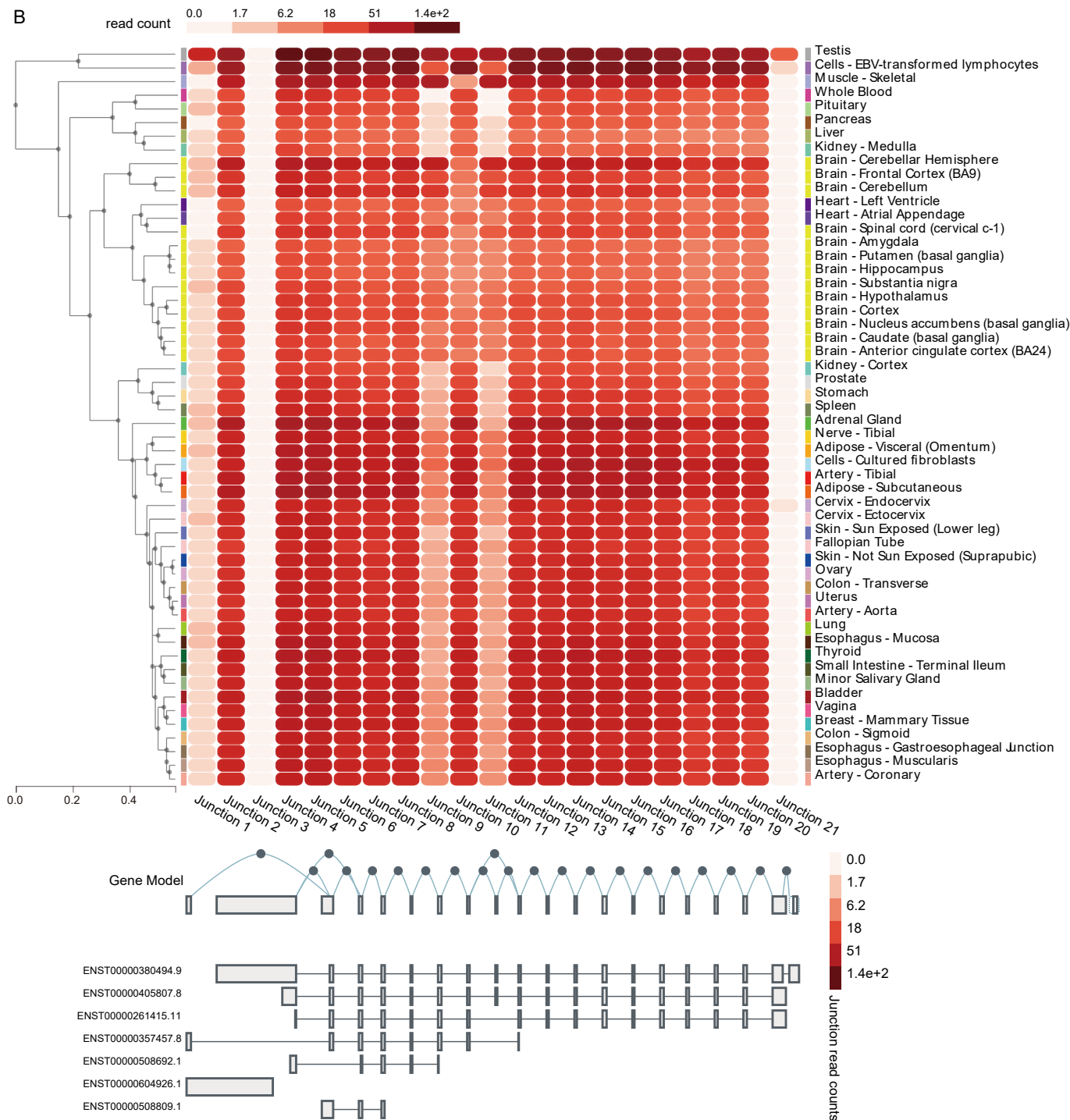

Supplement: S2 Fig — (A) Exon-level expression data of CERT1 (https://gtexportal.org/home/transcriptPage). The heatmap summarizes the median read counts per base of each exon across all tissues. This indicated that the transcript containing exon1 is barely detected in all tissues except for testis and Epstein-Barr virus-transformed lymphocytes. In most tissues, RNA-seq short reads from exon12 were detected at lower frequencies than those from exon11 or exon13. (B) Junction expression data of CERT1 (https://gtexportal.org/home/transcriptPage). The heatmap summarizes the median raw read counts of junctions from individual isoforms. This demonstrated that read counts of junction21 connecting exon1 with exon2 were barely detected, which indicated that isoform 3 of CERT1 is not expressed in most human tissues. The read counts of junction10 connecting exon11 with exon13 were detected at higher frequencies than junction9 or junction11, which indicated that the isoform 1 of CERT1 is the predominant transcript in most human tissues. (PDF) [file pone.0243980.s002.pdf]

S3 Fig

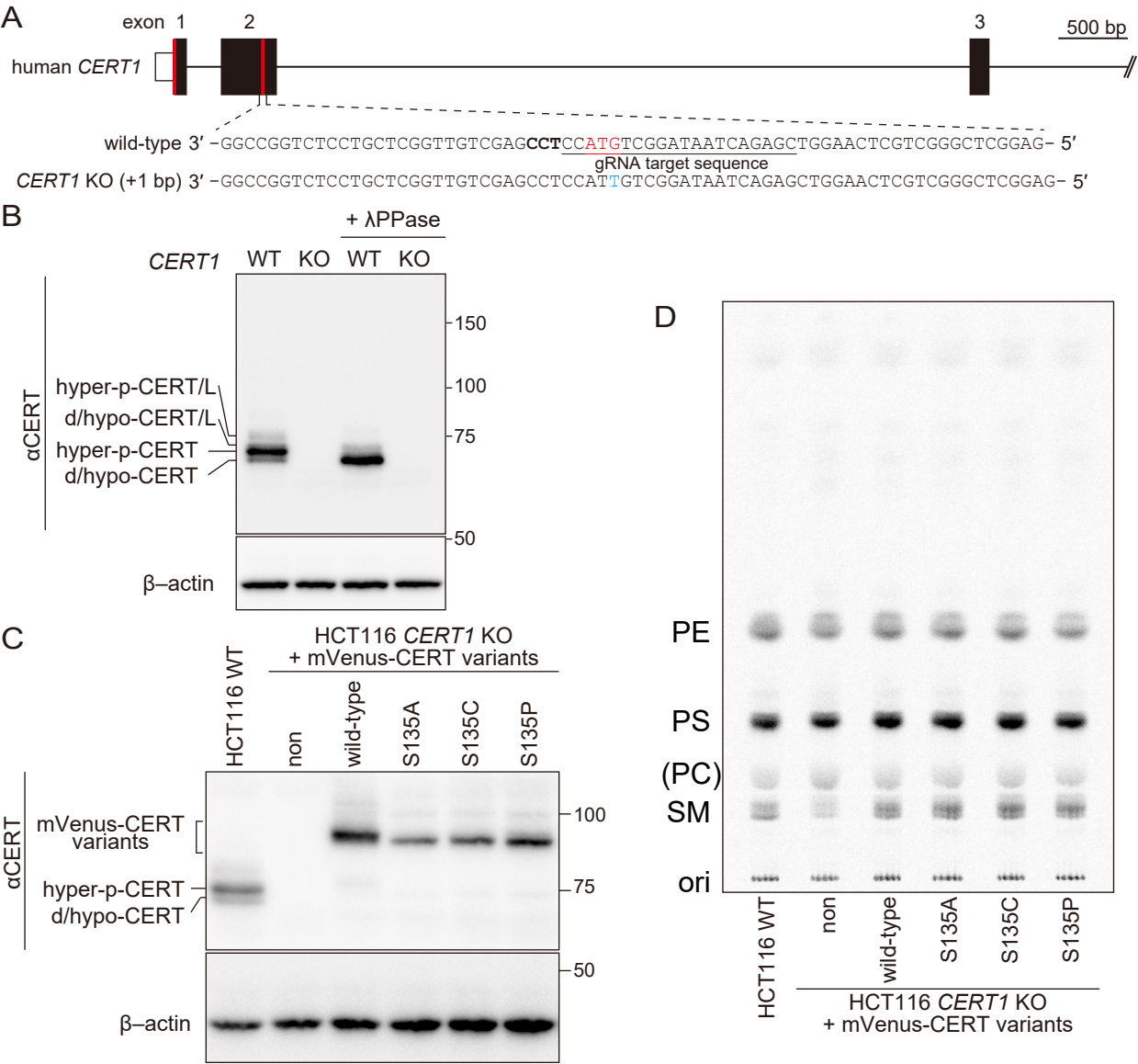

Supplement: S3 Fig — (A) Diagram of the location of gRNA-targeted sequences of human CERT1. The 5’-untranslated regions (UTRs) and coding regions are shown as white and black rectangles, respectively. The red lines represent the first and second methionines. In the WT allele, the protospacer adjacent motif (PAM) sequence is shown in bold. The red highlighted sequence (ATG) indicates the second methionine within exon2. The gRNA-targeted sequence is underlined. A 1 bp insertion that generates a frame-shift mutation is shown in blue in both CERT1 alleles from the CERT1 KO cell line. (B) Cell lysates prepared from WT and CERT1 KO HCT116 cells were incubated with or without λPPase and analyzed by Western blotting with the indicated primary antibodies. (C) WT and CERT1 KO HCT116 cells stably expressing various mVenus-CERT constructs were lysed and subjected to Western blotting analysis with the indicated primary antibodies. (D) WT and CERT1 KO HCT116 cells stably expressing the indicated mVenus-CERT variants were cultured with L-[U-14C]serine for 16 hr. Metabolically labelled lipids were separated by TLC analysis and visualized using an image analyzer. A representative image is shown. PC, putative phosphatidylcholine. Note that serine is directly incorporated into PS by the base exchange reaction with PC, and labelled PS is converted to PE by decarboxylation [34, 35]. Metabolic labeling of PC with L-[U-14C]serine can occur by two different pathways [34, 35]. One pathway occurs via N-methylation of labelled PE, and the other pathway occurs via the conversion of serine to pyruvate, which is then anabolized to fatty acids via acetyl-CoA, followed by the synthesis of PC from the labeled fatty acids. (PDF) [file pone.0243980.s003.pdf]

S4 Fig

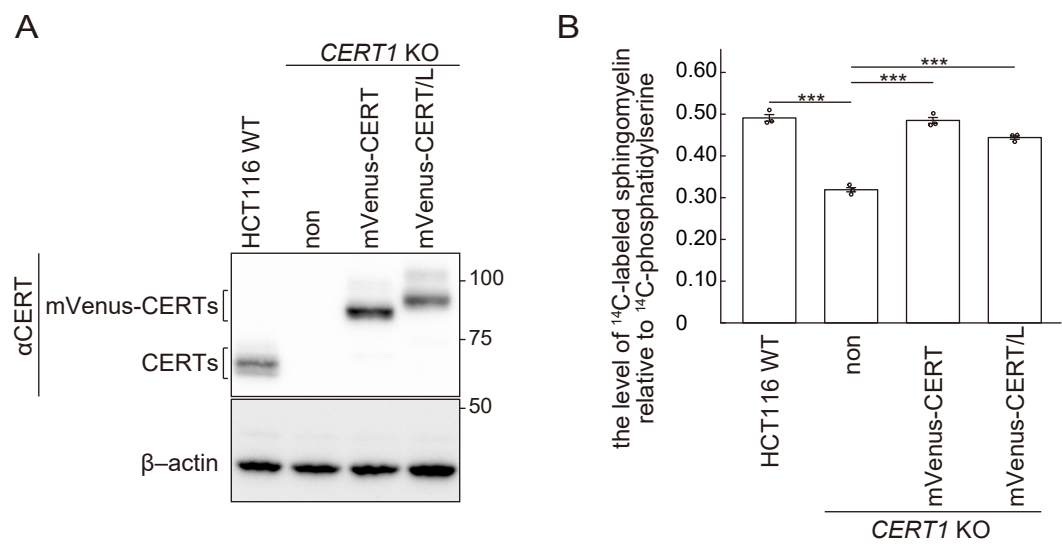

Supplement: S4 Fig — (A) WT and CERT1 KO HCT116 cells stably expressing either mVenus-CERT or mVenus-CERT/L were lysed and analyzed by Western blotting with the indicated primary antibodies. (B) The lipids from WT and CERT1 KO HCT116 cells stably expressing either mVenus-CERT or mVenus-CERT/L were metabolically labelled with L-[U-14C]serine. The labeled lipids were analyzed by TLC and visualized using an image analyzer. The data comprise the mean ± SEM; n = 3 (***, p < 0.001). (PDF) [file pone.0243980.s004.pdf]
