# Supplementary material for: Intellectual disability-associated gain-of-function mutations in CERT1 that encodes the ceramide transport protein CERT
Source: PLoS One. 2020 Dec 21;15(12):e0243980. doi: 10.1371/journal.pone.0243980 (PMC7751862; doi:10.1371/journal.pone.0243980)

Full unedited gel for Figure 3

3A αCERT

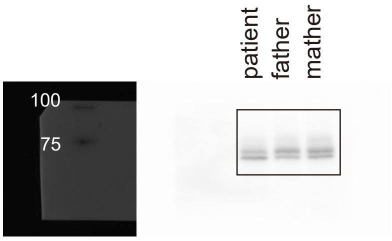

β-actin

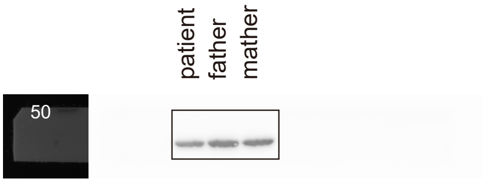

Full unedited gel for Figure 3

3B

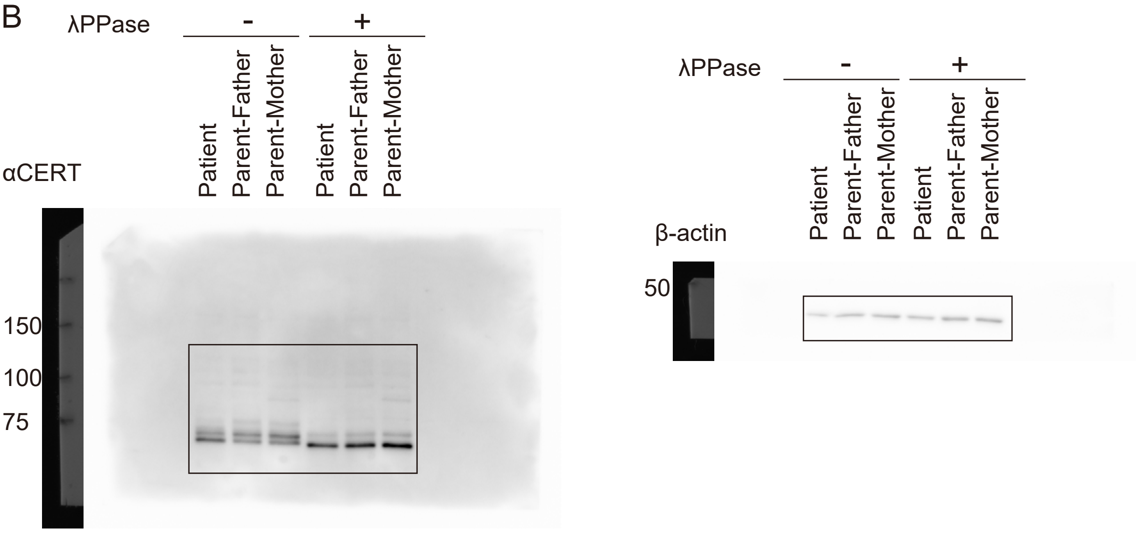

Full unedited gel for Figure 4

4A

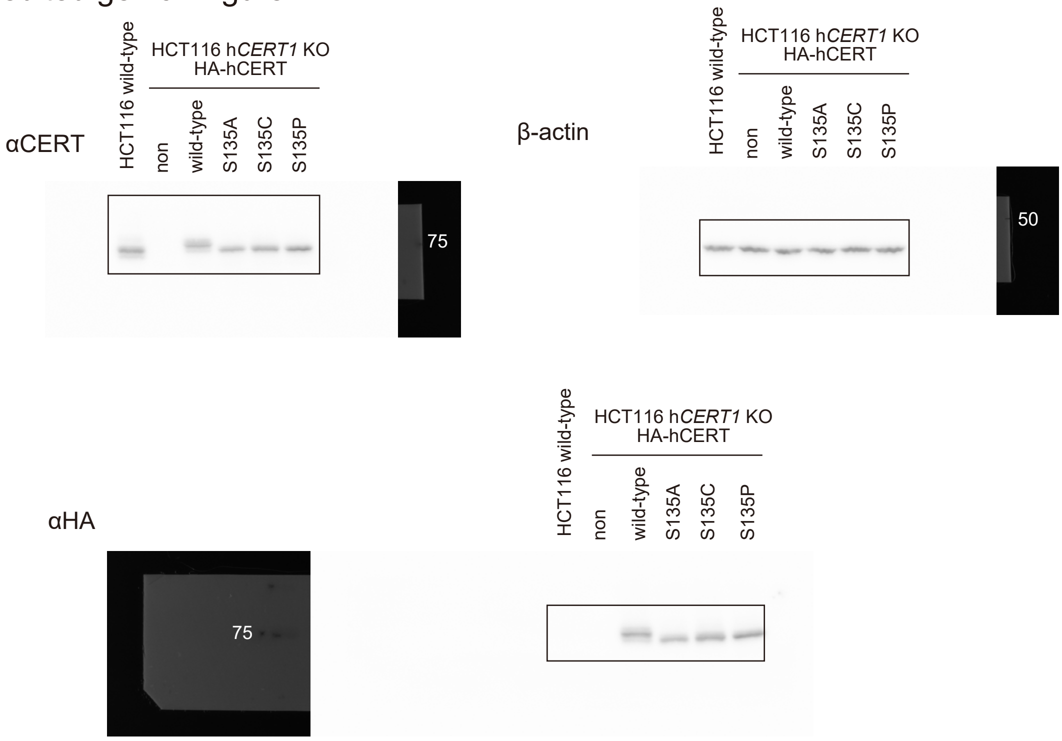

Full unedited gel for Figure 5

5A

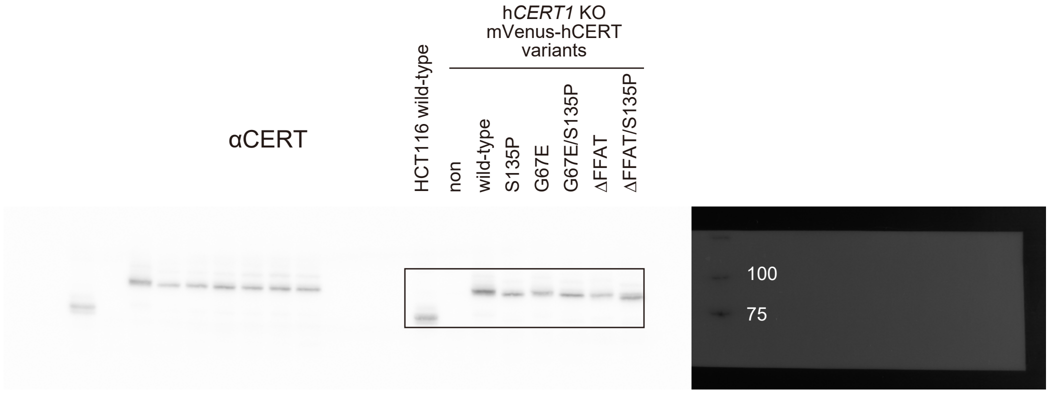

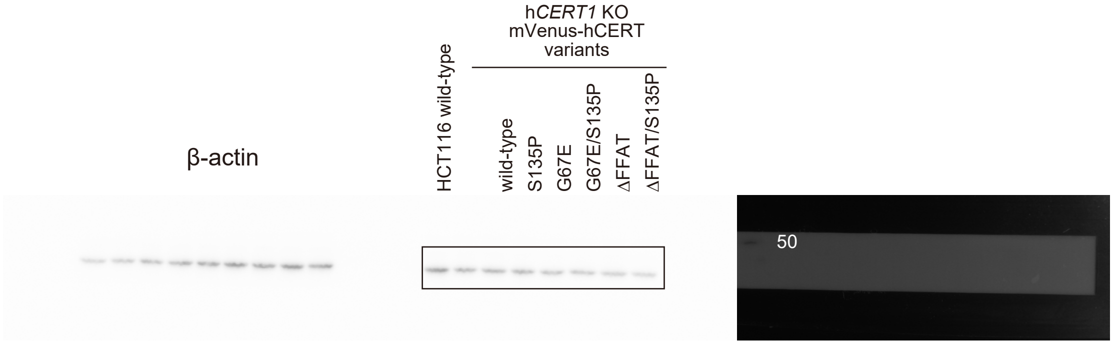

Full unedited gel for Supporting figure 3

3A

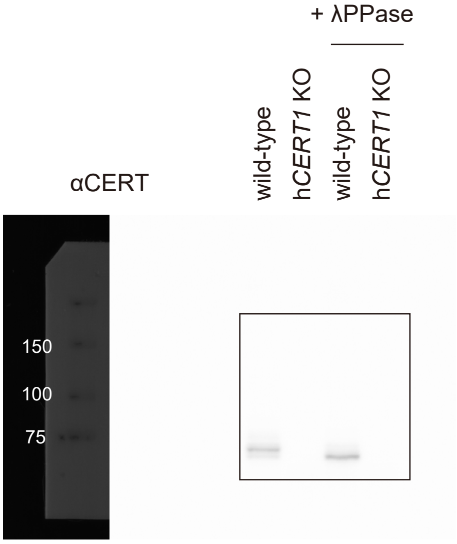

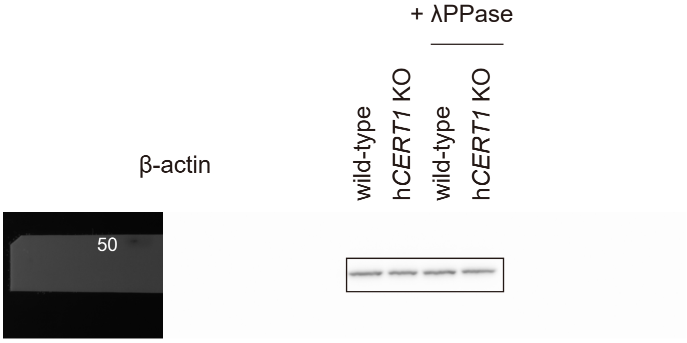

Full unedited gel for Supporting figure 3

3B

αCERT

HCT116 wild-type

h*CERT1* KO mVenus-hCERT variants

non
wild-type
S135A
S135C
S135P

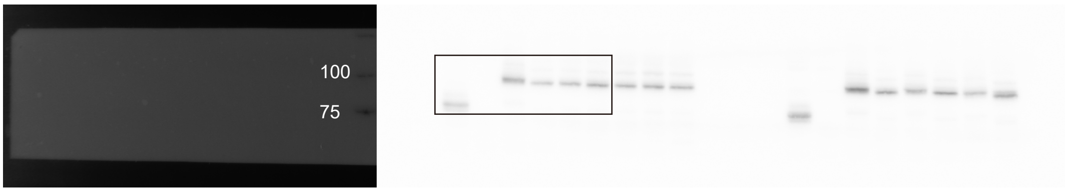

β-actin

HCT116 wild-type

h*CERT1* KO mVenus-hCERT variants

non
wild-type
S135A
S135C
S135P

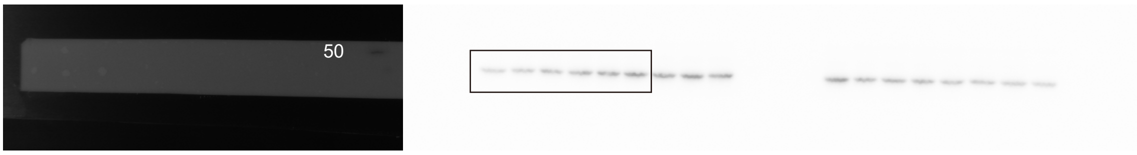

Full unedited gel for Supporting figure 4

4A

αCERT

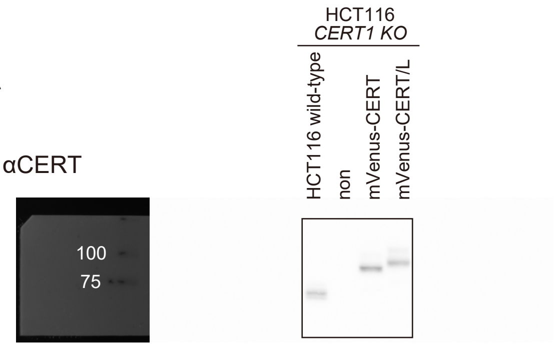

β-actin

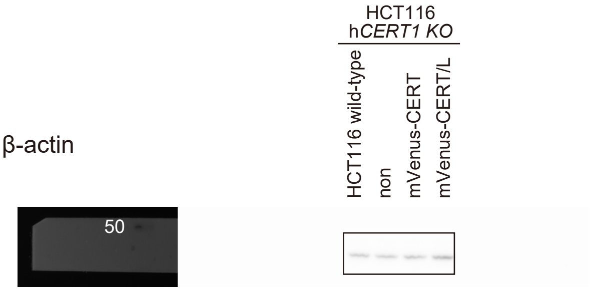

Supplement: S5 Fig — (PDF) [file pone.0243980.s005.pdf]
